# Solvent-Free Surface Engineering of Gas Diffusion Electrodes via Initiated Chemical Vapor Deposition for Durable Zinc–Air Batteries

**DOI:** 10.1021/acsomega.6c03441

**Published:** 2026-06-30

**Authors:** Gizem Ci̇hanoğlu, İklime Kayhan, Özgenç Ebi̇l

**Affiliations:** † Faculty of Engineering, Department of Chemical Engineering, 52972 Izmir Institute of Technology, Urla 35430, Izmir, Türkiye; ‡ ADVENST Energy Storage Systems, Gulbahce Mah., Kuluçka Merkezi Binası No: 1/45/11, Urla 35430, Izmir, Türkiye

## Abstract

Gas diffusion electrodes (GDEs) in rechargeable zinc–air (Zn-air) batteries often suffer from catalyst dissolution, electrolyte flooding, and degradation of the gas–liquid interface, which severely limit long-term cycling stability. Here, we report a solvent-free surface-engineering strategy based on initiated chemical vapor deposition (iCVD) to construct a dual-functional interfacial architecture within the GDE. A hydrophilic poly­(glycidyl methacrylate) (poly­(GMA)) interlayer was conformally deposited at the electrolyte interface to enhance electrolyte uptake and ionic transport, while a hydrophobic poly­(2-(perfluorohexyl)­ethyl acrylate) (poly­(PFHEA)) layer was introduced on the air side to preserve gas permeability. The optimized poly­(GMA) coating exhibited high electrolyte uptake (291 ± 8%) and enhanced ionic conductivity (1.61 mS cm^–1^), more than doubling that of the unmodified substrate. Incorporation of this interlayer within the GDE architecture reduced ohmic resistance and improved interfacial stability and transport characteristics, resulting in enhanced practical electrode performance. When integrated into Zn-air batteries, the modified electrode delivered a reduced charge–discharge voltage gap (0.734 V) and a round-trip efficiency of 60.3%. These findings demonstrate that vapor-phase polymer deposition provides a scalable route to engineer functional interfaces in GDEs, enabling improved stability and electrochemical efficiency in rechargeable Zn-air batteries. This work provides mechanistic insights into how vapor-deposited interfacial layers regulate ion transport and suppress catalyst degradation in gas diffusion electrodes.

## Graphical abstract

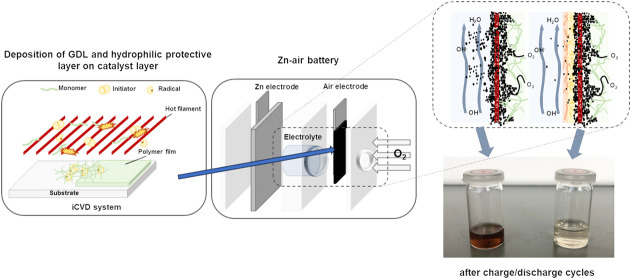

## 1 Introduction

The strong economic growth and expanding population are accompanied by an increase in energy demand, which is primarily met by fossil fuels such as oil, coal, and natural gas. According to the International Energy Agency, worldwide energy consumption is expected to increase by approximately 1.1% per year by 2030, and it is predicted to rise at a slower rate through to 2050.^1^ In recent years, European and global energy policies have focused on alternative, safe, competitive, and decarbonized energy technologies. Additionally, there is an urgent need to reduce greenhouse gas emissions and increase the share of renewable energy sources in total energy consumption. The variability inherent to renewable energy production has elevated the importance of advanced energy-storage systems, particularly rechargeable batteries, which are essential for maintaining a stable balance between energy supply and demand. Metal-air batteries have attracted significant attention as a possible alternative for electrical energy storage due to their high theoretical energy densities (1100–5200 Wh kg^–1^) and their lightweight compared to other battery technologies.^2–4^

Several metal-air chemistries are employing Al, Si, Mg, Fe, etc. as anode; however, zinc–air (Zn-air) batteries have been regarded as a promising candidate for the next-generation energy storage solution due to high theoretical energy density (1086 Wh kg^–1^), availability of anode materials, and inherent safety and nontoxicity compared to other rechargeable batteries, including lithium-ion batteries.^5,6^ However, rechargeable Zn-air batteries have not yet realized their full potential due to various issues associated with electrode reversibility, electrochemical stability, and limited cycle life.^7^ To address these primary challenges, research efforts have focused on zinc electrode and battery design,^8,9^ electrolyte additives,^10,11^ and electrocatalysts^12–15^ in Zn-air batteries to enhance battery performance. However, research on gas diffusion electrodes (GDEs) in Zn-air batteries has been limited.^16,17^ GDE is made of a porous hydrophobic membrane (Gas Diffusion Layer, GDL), a catalytic layer (CL), and an electrically conductive current collector, such as nickel and stainless-steel mesh. The cycling stability and lifetime of Zn-air batteries are significantly based on GDE performance. Catalyst poisoning, carbon corrosion, catalyst dissolution from catalyst layer to electrolyte,^18–20^ water intake from air, blockage and leakage of electrolyte (electrolyte flooding) after hundreds of charge/discharge cycles,^9,21^ insufficient oxygen input,^22,23^ oxygen transport limitations, and charge transport limitations^24,25^ are critical issues contributing to poor battery performance and shortened cycle life. Catalyst dissolution, particularly at high current densities during charging, remains the primary obstacle to achieving the theoretical performance of GDE, resulting in a reduction in the ionic conductivity of the electrolyte, a localized buildup of pressure within the air electrode, and, in turn, mechanical degradation of the air electrode. Another problem, electrolyte flooding, is caused by a slow penetration of electrolyte into the GDL, negatively affecting the oxygen reduction rate. To reduce the dissolution of the catalyst and restrict electrolyte flooding, different approaches with varying success have been tried, including impregnation of a proton conductor (such as Nafion) into the catalyst layer,^26,27^ use of hydrophobic/aerophilic and hydrophobic/hydrophilic layers, and reducing the thickness of GDEs.^28–31^ Zhang et al.^29^ fabricated an oxygen electrode with a hydrophilic/aerophobic layer in Zn-air flow batteries to enable the electrolyte to completely infiltrate the oxygen evolution reaction (OER) catalytic layer and prevent electrolyte leakage. The fabricated GDEs showed a 6% increase in energy efficiency compared to commercial electrodes. Moni et al.^30^ fabricated a silicon-based GDL for Zn-air batteries by tape casting. They also created an additional hydrophobic layer by coating the silicone layer with poly­(tetrafluoro ethylene) (PTFE). It was found that a double GDL with a total thickness of 390 μm significantly increased the oxygen transfer rate and achieved up to 200 charge/discharge cycles. Several strategies have been proposed to mitigate these limitations, including ionomer binders, graded hydrophilic–hydrophobic interfaces, and modified gas diffusion layer architectures. While these approaches provide partial improvements, they often suffer from inadequate barrier performance, poor long-term durability, or fabrication constraints related to solvent processing and limited control over film thickness. Most Zn-air batteries employ alkaline KOH electrolyte with or without additives. With increasing charge/discharge cycle counts, catalyst leaching into the electrolyte becomes an issue, as it has a direct impact on the cathode and overall battery performance. Therefore, preventing catalyst leaching into the electrolyte should be considered another key issue to be addressed. A proper high-performance GDE design should also consider a barrier layer to prevent the catalyst dissolution during battery operation. This barrier layer should preferably be hydrophilic and have a high electrolyte uptake capacity, excellent ionic conductivity, low electrocatalyst dissolution, and good durability. There are various methods in the literature for producing such a layer, including wet chemistry,^29^ solution deposition,^23^ nonsolvent-induced phase separation,^31^ and evaporation-deposition.^32^ However, these approaches still suffer from problems related to wetting and other solvent effects, as well as issues with thickness control and conformality.

Initiated chemical vapor deposition (iCVD) provides a unique, solvent-free fabrication platform that enables the production of conformal polymer films with precise control over thickness and chemistry, even within highly porous electrode architectures.^33^ iCVD enables functionalization of delicate substrates without clogging pores or altering catalyst morphology, making it particularly well suited for engineering interfacial layers inside GDEs.^34^ However, despite the growing use of iCVD in membrane science, surface engineering, and protective barriers, its application to GDE modification in Zn–air batteries has not yet been reported. We hypothesize that introducing a conformal, hydrophilic iCVD-derived interlayer at the electrolyte interface can simultaneously regulate electrolyte distribution, suppress catalyst dissolution, and stabilize the three-phase boundary during operation.

In this work, we propose a vapor-phase interfacial engineering strategy to address catalyst dissolution, electrolyte flooding, and transport limitations in Zn-air gas diffusion electrodes. Using iCVD, we construct a dual-layer architecture consisting of (i) a hydrophilic poly­(glycidyl methacrylate) (poly­(GMA)) interlayer intended to enhance electrolyte retention and ionic transport and (ii) a hydrophobic poly­(2-(perfluorohexyl)­ethyl acrylate) (poly­(PFHEA)) coating on the air-facing side to preserve gas-transport pathways and mitigate moisture ingress. In addition, a poly­(GMA)-coated polyester (PES) substrate is incorporated at the electrolyte interface as an ion-conductive barrier layer. This design enables simultaneous control over electrolyte wetting, interfacial stability, and gas transport within the GDE architecture. To the best of our knowledge, this study represents the first integration of an iCVD-deposited hydrophilic homopolymer interlayer into a Zn–air GDE structure. The resulting electrodes show improved ionic conductivity, reduced charge–discharge polarization, and lower catalyst leaching, highlighting the potential of solvent-free polymer thin-film engineering for advanced metal–air battery electrodes.

## 2 Materials and Methods

### 2.1 Chemical and Materials

Analytical-grade chemicals 2-(perfluorohexylethyl)­acrylate (PFHEA, Fluoryx Inc.) and glycidyl methacrylate (GMA, Sigma-Aldrich, 98%) and *tert*-butyl peroxide (TBPO, Sigma-Aldrich, 98%) were used during iCVD synthesis of polymer thin films as monomers and initiator, respectively. Potassium hydroxide (KOH, ISOLAB) was used as the electrolyte for testing electrolyte uptake, ionic conductivity, and electrochemical performance. A wet-laid nonwoven polyester (PES, 162 μm of thickness, 05TH-100 type, Hirose Substrate Manufacturing Co., LTD) and c-Si wafer were used as a substrate for various tests.

### 2.2 Fabrication of Polymer Coatings

Two different homopolymer films were deposited onto a commercial catalyst layer (CL, Gaskatel) via a custom-built iCVD system. Poly­(PFHEA) homopolymer was coated onto all catalyst layers to serve as GDLs only on the surface facing outside. The fluorinated monomer was heated to 65 °C in a stainless-steel container. Monomer vapor was metered into the chamber through a mass-flow controller (MFC) (MKS 1479A). TBPO was kept at room temperature and delivered to the reactor through an MFC (MKS 1479A). The PFHEA monomer and TBPO initiator were introduced into the chamber at flow rates of 0.39 and 0.13 sccm, respectively. A thickness of 800 ± 50 nm was targeted. Hydrophilic poly­(GMA) homopolymer coating was deposited on the inside surface of the commercial CL and onto the PES. For poly­(GMA) deposition, GMA monomer was vaporized in a stainless-steel container heated to 65 °C. The GMA monomer and TBPO initiator were introduced into the chamber using MFCs (MKS 1479A) at flow rates of 1.5 and 1 sccm, respectively. The average thickness of poly­(GMA) homopolymer films was 150 ± 50 nm. For both depositions, the filament temperature was set to 330 °C.

The effect of monomer partial pressure and substrate temperature on the deposition rate of poly­(GMA) homopolymer was investigated. In the first series of depositions (the pressure series, PGMA_1, PGMA_2, and PGMA_3), the reactor pressure was varied between 250 mTorr and 450 mTorr at a substrate temperature of 35 °C to investigate the influence of changing monomer partial pressure on the deposition rate. The second set of depositions (the substrate temperature series, PGMA_1, PGMA_4, and PGMA_5) was conducted by varying the substrate temperature from 25 to 35 °C, while keeping all other parameters constant. Table 1 summarizes the operating conditions used throughout the iCVD deposition study.

**Table 1:** Poly­(GMA) Film Deposition Experiment Conditions

**sample name**	**monomer**	**initiator**	***F***_**monomer**_ **(sccm)**	***F***_**initiator**_ **(sccm)**	***P***_**total**_ **(mTorr)**	***T***_**substrate**_ **(°C)**	***T***_**filament**_ **(°C)**
PGMA_1	GMA	TBPO	1.5	1	250	35	330
PGMA_2	GMA	TBPO	1.5	1	350	35	330
PGMA_3	GMA	TBPO	1.5	1	450	35	330
PGMA_4	GMA	TBPO	1.5	1	250	25	330
PGMA_5	GMA	TBPO	1.5	1	250	30	330

In this study, CLs incorporating the hydrophobic homopolymer poly­(PFHEA) are collectively referred to as GDEs. To enhance electrolyte wettability, we additionally examined GDEs coated with hydrophilic polymers using iCVD. This approach enables controlled modification of the interfacial region between the GDE and the aqueous electrolyte. The unmodified hydrophobic GDE containing only the poly­(PFHEA) homopolymer is denoted as b-GDE. When a poly­(glycidyl methacrylate) (poly­(GMA)) film is deposited onto the internal surfaces of the b-GDE via iCVD, the resulting sample is designated GDE_gma. A further sample, GDE_gma/PES, refers to a b-GDE containing the poly­(GMA) coating on the PES substrate. In both of GDE_gma and GDE_gma/PES samples, the GMA coating resides at the electrolyte interface. A schematic illustration of the GDE architectures described above is provided in Figure 1.

**Figure 1:**
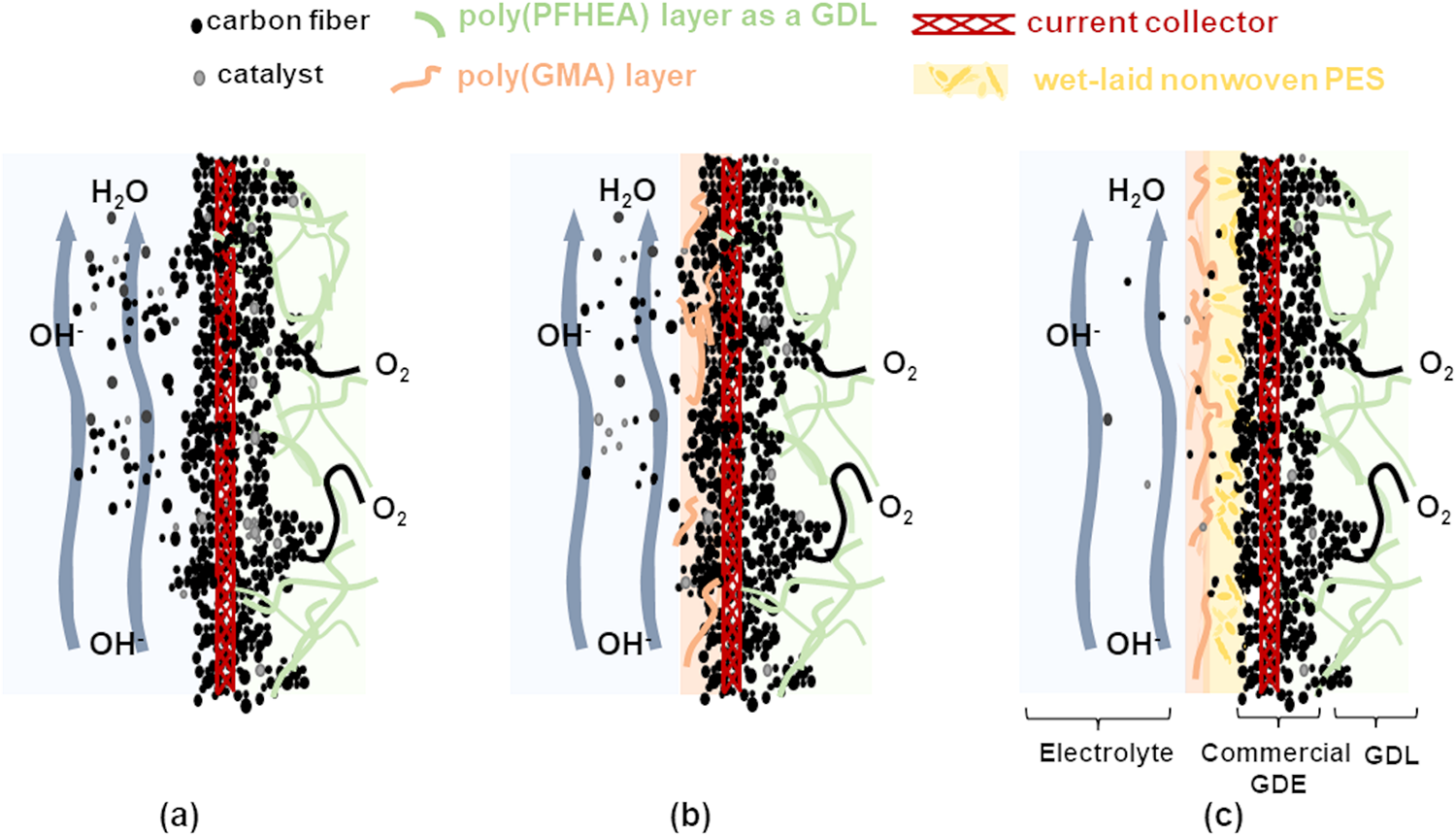
Schematic illustration for (a) b-GDE, (b) GDE_gma, and (c) GDE_gma/PES.

### 2.3 Characterization

The thickness of polymer films synthesized via iCVD was measured using an Mprobe-Vis20 reflectometer. Fourier Transform Infrared Spectroscopy (FTIR, PerkinElmer Inc.- Spectrum BX) was used to evaluate the quality and chemical composition of the polymer films. The spectra of the polymer films were measured from 4000 to 400 cm^–1^. All spectra were baseline-corrected and thickness-normalized. The microstructure of the coated films was investigated using a FEI Quanta 250 Scanning Electron Microscope (SEM). Contact angle measurements were conducted to investigate the hydrophilicity and hydrophobicity of films using a Theta Optical Tensiometer. Static contact angle measurements were performed in 2 s by dropping 5 μL of ultrapure water onto the coated surfaces. The hydrophilicity of poly­(GMA) homopolymers and the wet-laid nonwoven PES were determined using the electrolyte uptake measurements. The liquid electrolyte uptake was evaluated using the following equation:

$$uptake\;\left(\%\right)=\left[\frac{W-W_{o}}{W_{o}}\right]\times 100$$

where *W* and *W*_o_ indicate the weights of substrate soaking into 6 M KOH solution for liquid electrolyte after 24 h and dry substrate, respectively.

### 2.4 Testing of GDEs

A potentiostat/galvanoostat/ZRA (Gamry model 22162) with a tripolar electrode measurement apparatus was used for all electrochemical measurements. Electrochemical tests were performed in a 3-electrode FlexCell-PP system (Gaskatel GmbH) with 6 M KOH as the electrolyte, a Pt wire as the counter electrode, and a hydrogen electrode (RHE) as a reference electrode at 25 ± 2 °C. b-GDE, GDE_gma, and GDE_gma/PES were used as working electrodes. Linear sweep voltammetry (LSV) was performed between 0.3 and 1.8 V at a scan rate of 10 mV s^–1^ in 1 M KOH. The open-circuit voltage (OCV) of GDE is monitored for 30 min to allow the electrode/electrolyte interface to reach a stable equilibrium. AC impedance measurements of half-cells were also carried out. Ionic conductivity was evaluated by electrochemical impedance spectroscopy (EIS) in the frequency range from 10 kHz to 10^–1^ Hz at room temperature. Ionic conductivity (σ, mS cm^–1^) was calculated by the following equation:

$$\sigma =\frac{L}{\mathrm{RA}}$$

where *L* is the hydrophilic layer thickness (cm), *A* is the area of the sample (cm^2^), *R* is the resistance of the hydrophilic layer (ohmic resistance) (Ω), which can be estimated from the Nyquist plot obtained from the EIS.

For aging test, galvanostatic charge–discharge cycling was performed at 10 mA cm^–2^ in 1 M KOH using 20 min charge and 20 min discharge steps for 30 cycles, integrated with pre- and postaging EIS measurements conducted at selected discharge and charge potentials (0.3, 0.9, 1.6, and 1.7 V vs RHE) to track the electrochemical evolution. The resulting impedance spectra were fitted using equivalent circuit models. These analyses were performed to elucidate degradation mechanisms associated with prolonged cycling and to evaluate the role of the GMA-PES interlayer in mitigating aging-related transport and stability losses.

Following cycling tests, the dissolution of the catalyst through the electrode to the electrolyte was investigated by an Inductively Coupled Plasma-Optical Emission Spectrometer (ICP-OES) (Agilent 7500ce Octopole) on b-GDE and GDE_gma/PES samples.

The performance of Zn-air batteries manufactured using modified GDEs was evaluated using a battery testing system (NEWARE, CT-4008Tn-5 V6A-S1, China). The cycling test of the battery was conducted at room temperature with a fixed charge–discharge current of 5 mA cm^–2^. The charge and discharge cutoff voltages were set to 2.4 and 0.7 V, respectively.

## 3 Results and Discussion

Poly­(GMA) coatings were obtained by varying monomer partial pressure and substrate temperature during polymerization via iCVD. The deposition rate of poly­(GMA) depends on the concentration of adsorbed monomers on the substrate surface.^34,35^ The surface concentration of a monomer depends on its partial pressure and substrate temperature, as seen in Figure 2. For the poly­(GMA) homopolymer coatings, higher deposition rates were observed at lower substrate temperatures and higher monomer partial pressures. The low substrate temperature enhances monomer adsorption on the surface, resulting in a higher deposition rate.^35,36^

**Figure 2:**
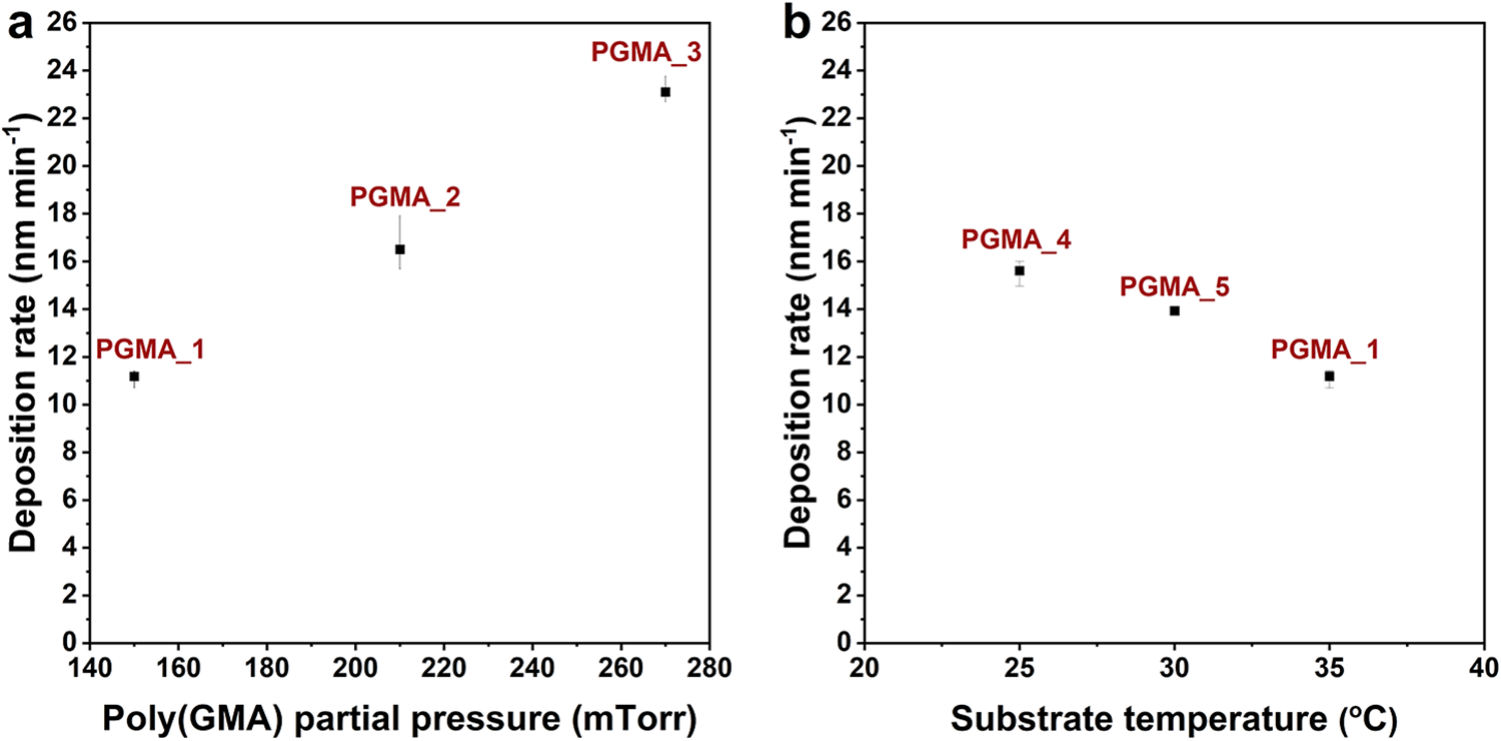
Deposition rate as a function of (a) poly­(GMA) partial pressure, and (b) substrate temperature.

Chemical compositions of pristine PES, poly­(GMA) homopolymers on c-Si wafer and PGMA_1 coated PES were investigated by FTIR analysis. The structure of poly­(GMA) was shown in Figure 3. There is no observable difference for FTIR peaks between pristine PES and pGMA_1 coated PES due to the thin film (∼150 nm), as shown in Figure 4. For PGMA_1 homopolymer, the peaks at 758, 845, and 906 cm^–1^ in the FTIR spectrum represent C–O–C epoxy ring vibrations and the peaks at 840–853 cm^–1^, 912–920 cm^–1^, and near 1241–1251 cm^–1^ show the symmetric epoxy ring deformation, asymmetric epoxy ring deformation, and the epoxy ring breathing vibration, respectively.^37–40^

**Figure 3:**
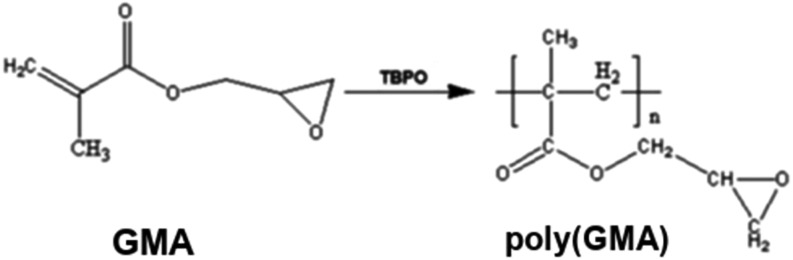
Structures of GMA monomer and poly­(GMA).

**Figure 4:**
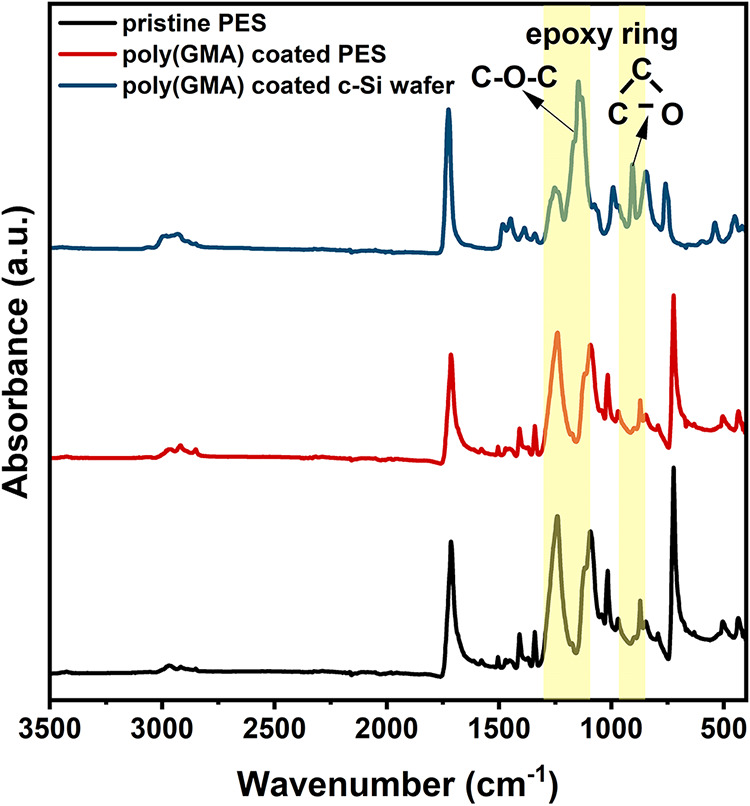
FTIR spectra of poly­(GMA) coated c-Si wafer and PES, and pristine PES.

Figure 5 shows the surface morphology of pristine PES and poly­(GMA) coated PES with varying substrate temperature and monomer partial pressure. The change in process parameters (substrate temperature and partial pressure) can affect the surface properties (conformability, smoothness etc.) of deposited films. Conformality of the depositions, which is critical for achieving favorable surfaces, can be enhanced by operating at high substrate temperatures or low partial pressures, thereby reducing the monomer concentration on the substrate surface.^37^ SEM images showed mostly conformal coatings on the surface of the substrates. The effect of deposition conditions on the quality of conformality and film morphology can be seen in Figure 5a–d, with insets showing higher magnification. While process conditions leading to higher deposition rates tend to result in slightly higher amounts of nonconformity (and slightly thicker coatings), no significant differences were observed. These observations suggest that deposition conditions used in this study did not result in significant differences in surface morphology.

**Figure 5:**
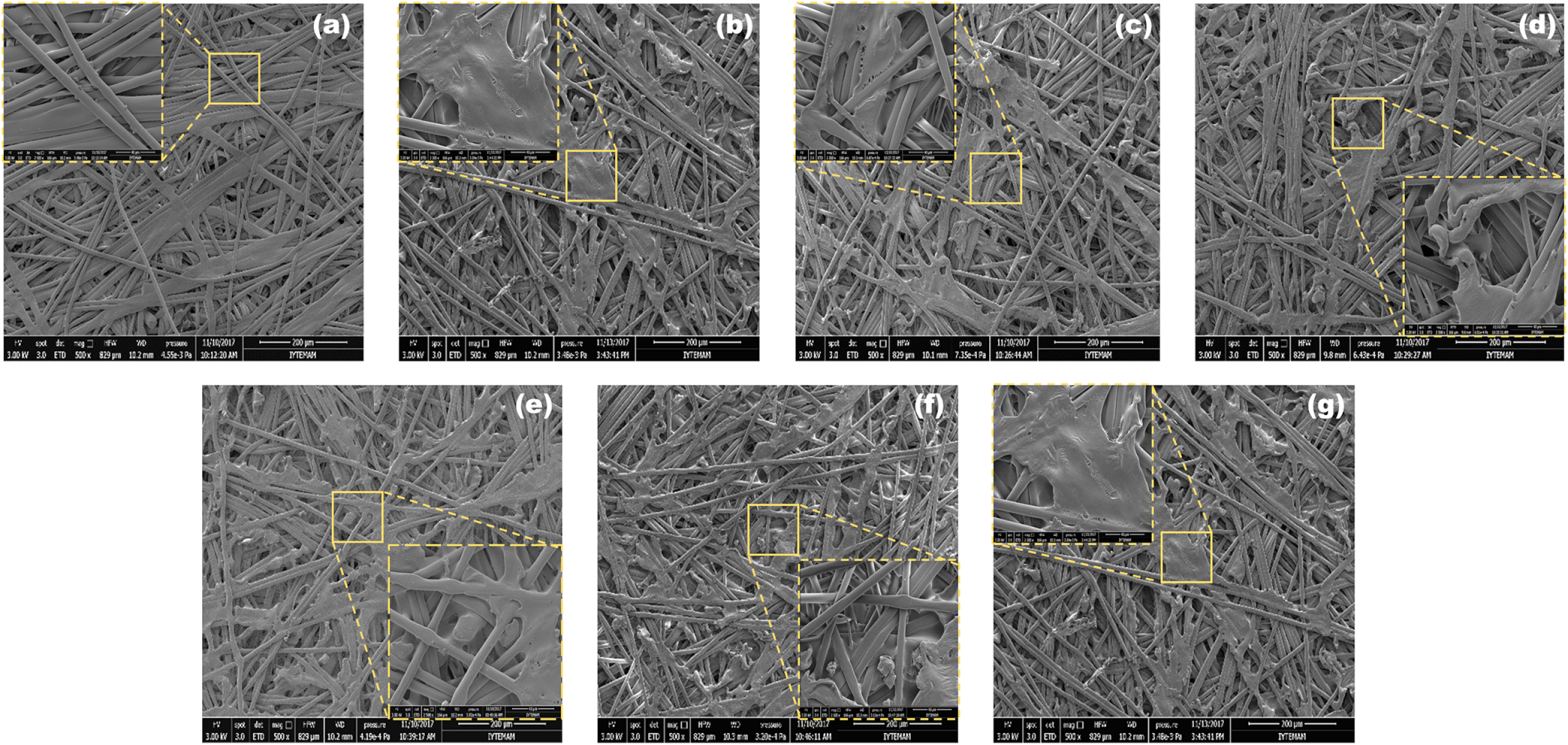
SEM images of (a) pristine PES and (b,g) PGMA_1, (c) PGMA_2, (d) PGMA_3, (e) PGMA_4, and (f) PGMA_5 coated PES samples.

A hydrophilic coating should have high electrolyte wettability between the CL and the electrolyte to increase the efficiency of the oxygen reduction reaction (ORR) and oxygen evolution reaction (OER). Additionally, the hydrophilic layer is expected to prevent the catalysts from migrating into the electrolyte and disrupting the OH^–^ concentration balance during charge/discharge cycles. The contact angle measurement can be used to evaluate the affinity between the electrolyte and the hydrophilic protective layer. The contact angle values of the bare substrate and poly­(GMA) coated substrates were measured and listed in Table 2. The contact angle of the PGMA_1-coated substrate was lower than that of other substrates deposited at different partial pressures (CA = 75 ± 2–77 ± 2°). When compared to poly­(GMA) coated substrates, there was no observable difference in their contact angles when the substrate temperature was changed during deposition (CA = 72 ± 1–77 ± 1°).

**Table 2:** Contact Angle Measurements of Uncoated and Poly­(GMA) Coated Substrates

	**samples**					
	**bare**	**PGMA_1**	**PGMA_2**	**PGMA_3**	**PGMA_4**	**PGMA_5**
**contact angle**°	65 ± 2	67 ± 1	75 ± 2	77 ± 2	72 ± 1	77 ± 1

The electrolyte wettability was investigated via electrolyte uptake measurements. The electrolyte uptake of the substrate as a function of deposition conditions (monomer partial pressure and substrate temperature) in the iCVD system was analyzed over 24 h. As shown in Figure 6, the electrolyte uptake of poly­(GMA) coated PES was better than that of pristine PES (248 ± 9%) due to hydrophilic functional groups (ester group, -RCOOR) of poly­(GMA). The PGMA_1 coated PES (291 ± 8%) also showed a larger electrolyte uptake capability compared to samples deposited at different partial pressures (261 ± 14% for PGMA_2 and 255 ± 11% for PGMA_3). Additionally, the PGMA_1-coated PES exhibited better wettability compared to other ones deposited at various substrate temperatures (250 ± 9% for PGMA_4 and 280 ± 11% for PGMA_5). The wettability of polymer coatings is dependent on their conformability; therefore, changes in deposition conditions of the iCVD system can affect film wettability.^38^ Based on this observation, the poly­(GMA) coating deposited at a monomer partial pressure of 150 mTorr and a substrate temperature of 35 °C (PGMA_1) was selected as the primary sample for comprehensive electrochemical testing.

**Figure 6:**
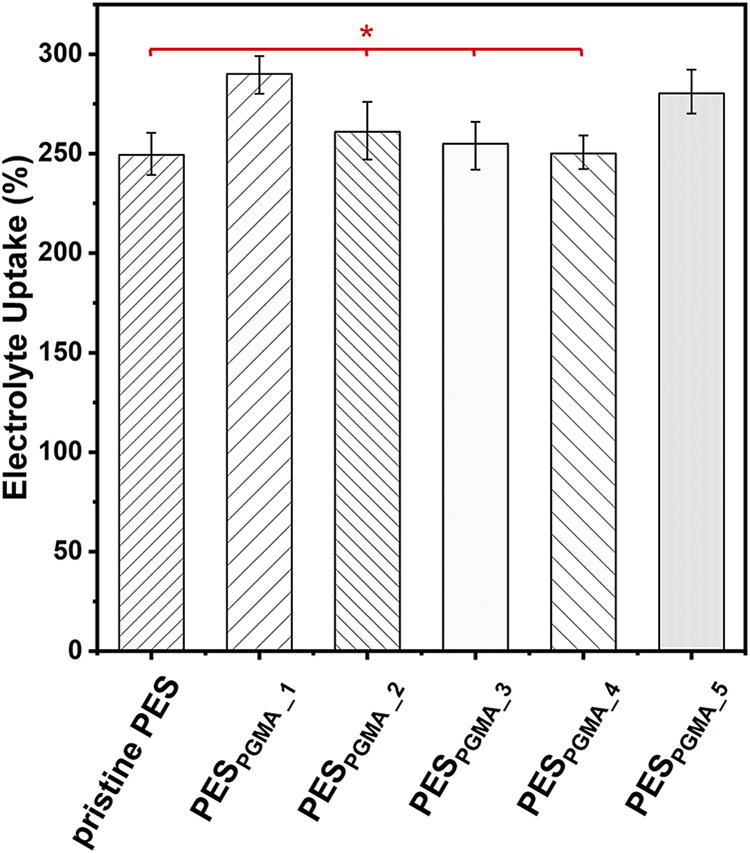
Electrolyte uptake results for pristine PES and poly­(GMA) coated PES (one asterisk (*) denotes a *p* ≤ 0.05 significance between samples).

The enhancement of electrochemical performance partially relies on increased electrolyte uptake and higher ionic conductivity.^41^ Electrochemical impedance spectroscopy (EIS) was therefore used to compare the ohmic resistance of b-GDE and GDE_gma/PES. The ohmic resistance of GDE_gma/PES (1.43 Ω) was smaller than that of b-GDE (3.22 Ω), while the ionic conductivity of GDE_gma/PES (σ = 1.61 mS cm^–1^) was 2.3 times higher than that of b-GDE (σ = 0.71 mS cm^–1^). The ohmic resistance and ionic conductivity of the GDE_gma sample were also evaluated as 2.83 Ω and 0.82 mS cm^–1^, respectively. However, it should be noted that this configuration exhibited higher variability and less stable impedance responses compared to b-GDE and GDE_gma/PES. Therefore, while the values are reported here for reference, the discussion primarily focuses on the more reproducible systems to ensure reliable comparison. The increase in ionic conductivity suggests more effective ionic transport arising from improved electrolyte wetting within the porous structure. This behavior is consistent with the presence of hydrophilic ester functionalities in the GMA-containing layer. This improvement can be attributed to the formation of continuous ion-conductive pathways enabled by the hydrophilic GMA network, which facilitates electrolyte penetration while minimizing local ionic resistance within the porous structure.

In addition to the hydrophilic poly­(GMA) layer at the electrolyte interface, the hydrophobic poly­(PFHEA) coating on the air-facing side plays a critical role in maintaining gas transport pathways by preventing excessive moisture accumulation and electrolyte intrusion into the gas diffusion layer. This behavior is consistent with our previous work, where PFHEA-based fluorinated coatings were shown to exhibit high oxygen permeability and strong hydrophobicity, enabling efficient oxygen transport while restricting moisture uptake in gas diffusion layers.^34^ The fluorinated nature of PFHEA provides strong hydrophobicity, which stabilizes the three-phase boundary and minimizes flooding-related transport limitations. Although this layer does not directly enhance catalytic activity, it contributes to the overall electrode performance by preserving oxygen accessibility during operation. This complementary function highlights the importance of decoupling gas transport and electrolyte management within the GDE architecture.

The electrochemical performance of the prepared electrodes (b-GDE, GDE_gma, and GDE_gma/PES) was evaluated using LSV and OCV measurements (Figure 7). As shown in Figure 7a, the GDE_gma/PES electrode exhibits the highest current density across the investigated potential range for OER, indicating more favorable overall electrode performance than b-GDE under these conditions. This response is consistent with improved electrolyte accessibility and reduced interfacial resistance provided by the GMA-containing/PES architecture. Figure 7b presents the ORR polarization behavior. Although the modified electrodes do not uniformly outperform b-GDE, the overall polarization response suggests that interfacial wetting and transport effects contribute substantially to the observed full-electrode behavior. In particular, the response of GDE_gma/PES at higher potentials is consistent with a more stable three-phase interface and improved ionic access during operation.

**Figure 7:**
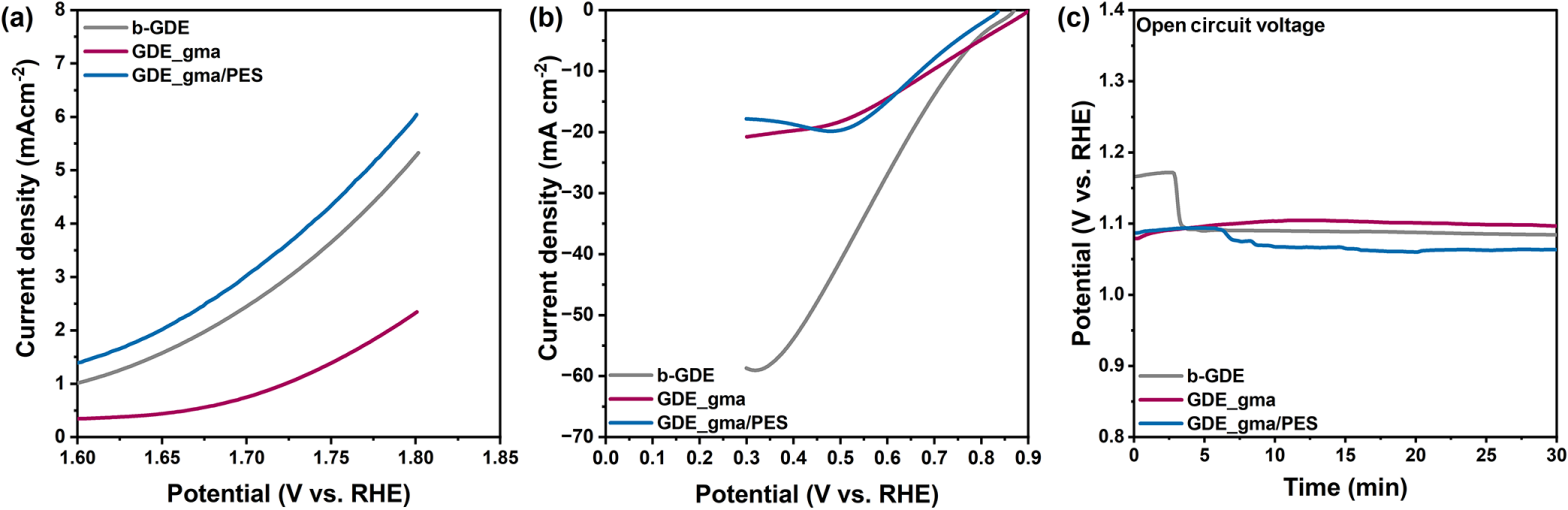
LSV polarization curves for (a) OER, (b) ORR, and (c) open circuit voltage for b-GDE, GDE_gma, and GDE_gma/PES.

OCV measurements reveal that the modified electrodes maintain more stable potentials during the 30 min monitoring period compared to the bare electrode, as shown in Figure 7c. The relatively stable OCV values observed for GDE_gma and GDE_gma/PES suggest improved electrode/electrolyte interfacial stability and structural robustness. Additionally, this observation supports the idea that the polymer coatings create a trade-off between intrinsic catalytic activity and interfacial stability. Slight losses in ORR kinetics are effectively compensated by enhanced electrode durability and improved interfacial transport. To further analyze the reaction kinetics underlying these observations, Tafel analysis was performed. Therefore, the primary advantage of the GDE_gma/PES architecture should not be interpreted as enhanced intrinsic electrocatalytic activity, but rather as improved resistance against interfacial degradation and aging-related transport losses during prolonged operation.

The effect of the coatings on the electrochemical performance of the GDEs was further examined using Tafel analyses for both ORR and OER (Table 3). In principle, a lower Tafel slope reflects more favorable reaction kinetics and reduced polarization.^42–44^ However, the Tafel slopes in Table 3 do not show a uniform improvement for all modified electrodes relative to b-GDE. For ORR, GDE_gma/PES (71.2 mV dec^–1^) shows a modest improvement relative to GDE_gma (73.8 mV dec^–1^), but b-GDE exhibits the lowest ORR slope overall (58.5 mV dec^–1^). Likewise, the higher OER slope measured for GDE_gma/PES indicates that the performance gains observed during cycling cannot be attributed solely to faster intrinsic electrocatalytic kinetics. Instead, these observations suggest that electrolyte management, interfacial stability, and suppression of degradation processes play a central role in the improved cell-level behavior of GDE_gma/PES. These findings clearly indicate that the improved cycling performance is decoupled from intrinsic catalytic activity and instead governed by interfacial and transport-related effects.

**Table 3:** Tafel Slopes for ORR and OER

**sample**	**OER**	**ORR**
**b-GDE**	77.5	58.5
**GDE_gma**	77.3	73.8
**GDE_gma/PES**	99.8	71.2

The lower ORR activity observed for the modified electrodes compared to b-GDE can be attributed to the additional transport resistance introduced by the conformal polymer layers. Such behavior has been widely reported in gas diffusion electrodes and catalyst-coated systems, where protective or functional interlayers may partially hinder reactant diffusion despite improving overall electrode stability.^44,45^ In particular, previous studies have shown that the introduction of polymeric or ionomeric layers can reduce apparent catalytic activity due to mass transport limitations, even when long-term performance is improved.^26,27^ For example, binder-rich catalyst layers or hydrophilic coatings are known to introduce diffusion resistance for oxygen, thereby affecting ORR polarization behavior.^44,46^ However, these layers enhance electrolyte distribution, maintain effective triple-phase boundaries, and suppress catalyst degradation, which become dominant factors under operating conditions.^26,45^ Therefore, the results of this study are consistent with the established understanding that there exists a trade-off between intrinsic catalytic kinetics and interfacial stability in modified gas diffusion electrodes.^45,46^ The improved overall electrode performance observed for GDE_gma/PES is thus primarily governed by enhanced interfacial transport and structural stabilization rather than intrinsic catalytic activity.

Overall, the Tafel analysis indicates that the improved cycling behavior of GDE_gma/PES does not originate from enhanced intrinsic electrocatalytic kinetics. Instead, the improved performance is primarily associated with interfacial stabilization, improved electrolyte distribution, and suppression of degradation processes.

The enhanced electrochemical performance of GDE_gma/PES is therefore more reasonably attributed to reduced interfacial losses and improved stability during operation than to a simple increase in intrinsic catalytic activity. The higher current density observed for GDE_gma/PES at similar potentials is consistent with facilitated ionic transport, improved electrolyte distribution, and better retention of the electrochemically active architecture during measurement. Such behavior is commonly associated with improved mass transport and lower degradation in modified gas diffusion electrodes.^45,46^

Aging tests of the GDE were further expanded through combined galvanostatic durability testing, pre- and post-aging test electrochemical impedance spectroscopy (EIS), and metal dissolution analysis to elucidate the degradation mechanisms governing long-term electrode performance. Figure 8 illustrates the cycling behavior over 30 cycles at 10 mA cm^–2^ in 1 M KOH electrolyte using alternating 20 min charge/discharge steps. Although both electrodes initially exhibited comparable discharge potentials, the bare electrode underwent rapid deterioration after prolonged cycling, whereas the GDE_gma/PES architecture maintained significantly improved electrochemical stability throughout the test period. In particular, the GDE_gma/PES electrode preserved a relatively stable discharge response up to approximately the 25th cycle. At the same time, the bare electrode exhibited continuous polarization growth and severe discharge-voltage decay after the 15th cycle. Interestingly, the charging potential gradually decreased from approximately 1.72 to 1.63 V and subsequently stabilized during cycling, suggesting gradual evolution toward a more stable electrode/electrolyte interface during prolonged operation. These findings indicate that the GMA-PES interlayer effectively mitigates degradation pathways associated with electrolyte flooding and catalyst deterioration, resulting in more sustained electrochemical performance.

**Figure 8:**
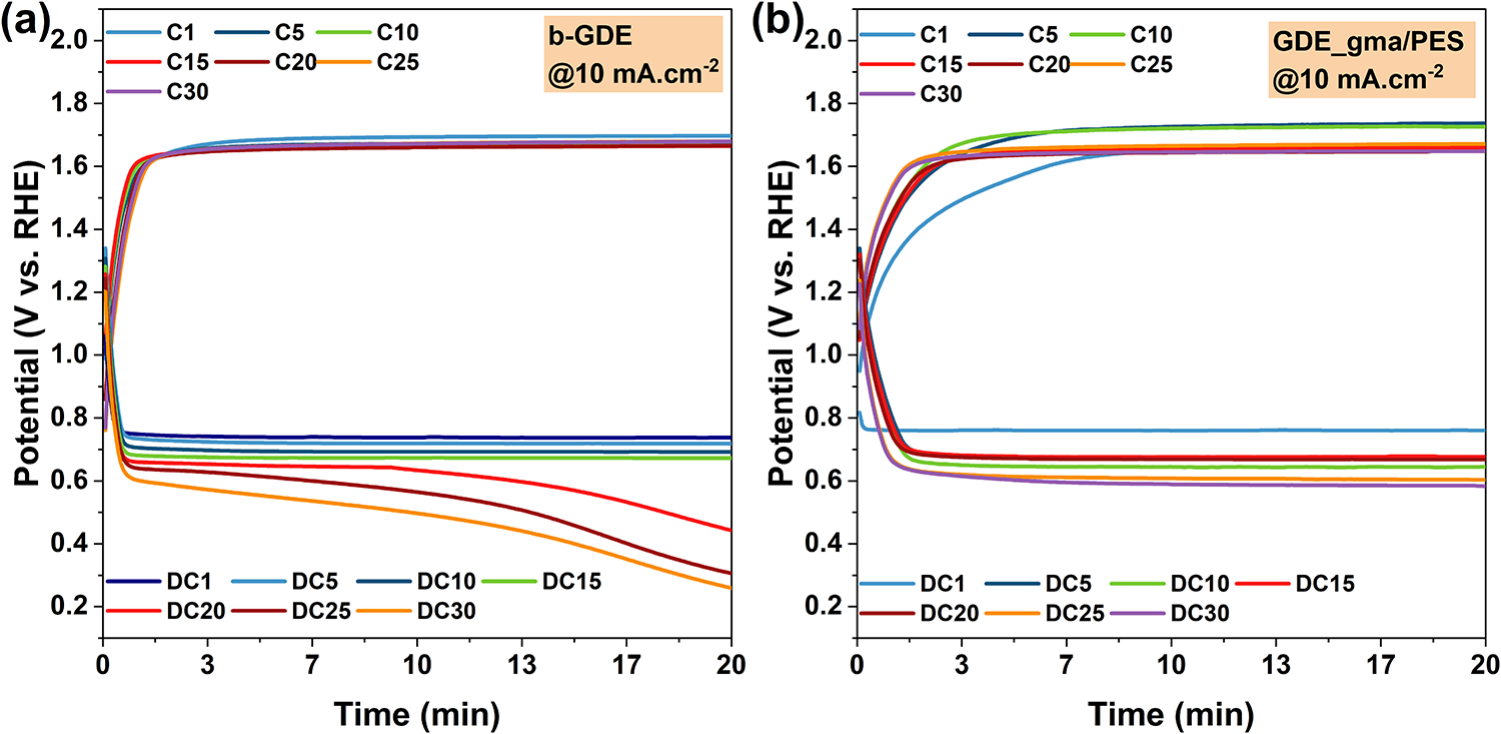
Aging/durability evaluation of (a) b-GDE and (b) GDE_gma/PES electrodes during galvanostatic charge–discharge cycling in 1 M KOH at a current density of 10 mA cm^–2^, using alternating 20 min charge and 20 min discharge steps.

To further elucidate the mechanisms governing this performance, Electrochemical Impedance Spectroscopy (EIS) measurements were conducted at specific potentials (0.3, 0.9, 1.6, and 1.7 V vs RHE) both before and after the 30-cycle durability test (Figure 9 and Table 4). The corresponding Nyquist plots, recorded from 10 kHz to 0.1 Hz, were fitted using the equivalent circuit models (ECM) presented in Figure S1 (in Supporting Information). The stability of the ohmic resistance (*R*_1_ ≈ 1.2 Ω) across all measurements indicates preserved ionic and structural integrity of the GMA-PES interlayer. At discharge potentials (0.3 and 0.9 V), the charge-transfer resistance (*R*_2_) increased significantly after cycling, accompanied by the appearance of a Warburg diffusion element, suggesting increased oxygen transport limitations and flooding-related degradation during prolonged discharge operation. In contrast, the charging response exhibited lower interfacial resistance after cycling, with *R*_2_ decreasing from 6.92 to 2.60 Ω at 1.6 V and from 2.61 to 1.14 Ω at 1.7 V. This behavior is consistent with the lower charging overpotentials observed during galvanostatic cycling and suggests improved electrolyte accessibility at the electrode interface. The increase in the constant phase element (CPE) exponent values after cycling additionally indicates the formation of a more homogeneous and electrochemically accessible interface. The increase in a_1_ parameter further indicates the evolution toward a more electrochemically accessible interface after prolonged operation.

**Figure 9:**
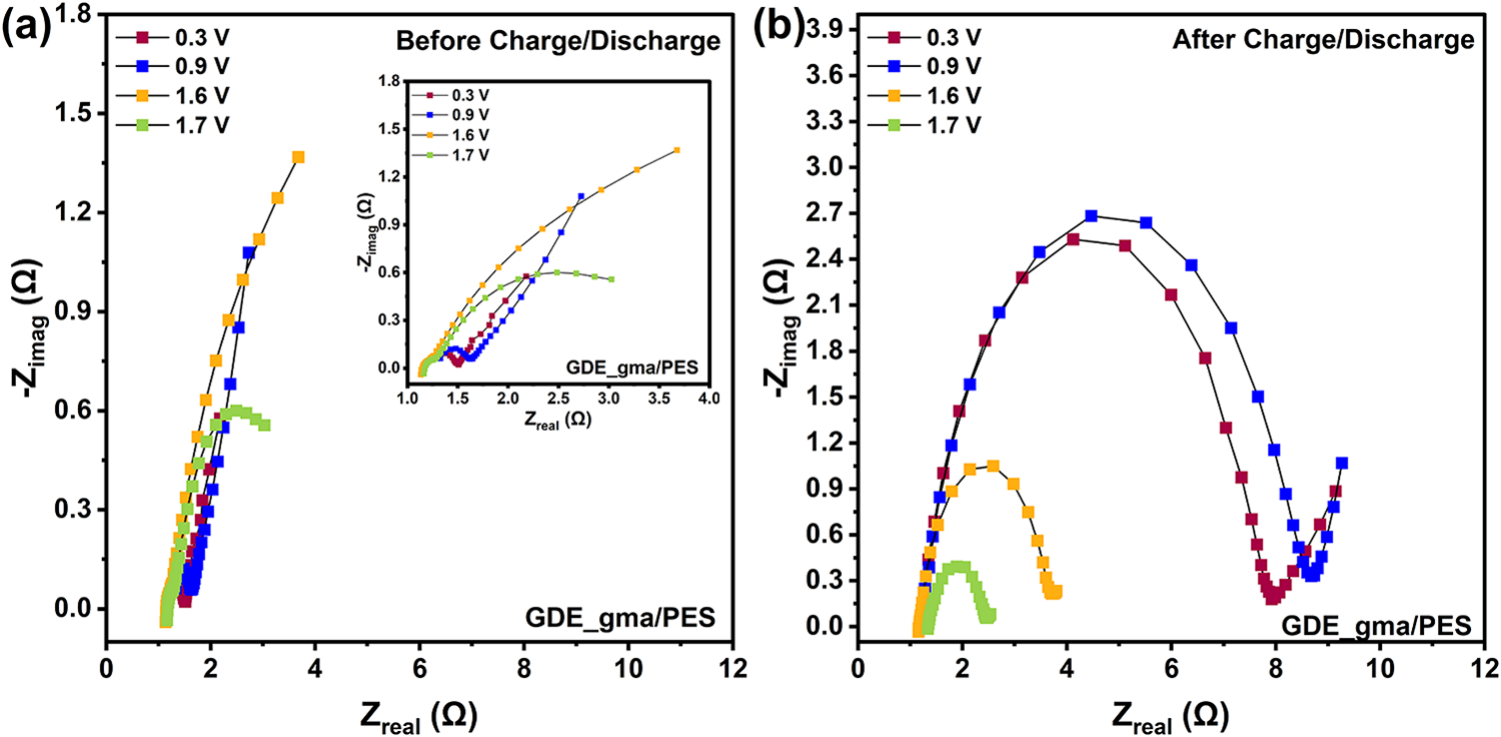
Pre- and post-aging EIS analysis of GDE_gma/PES recorded at representative discharge and charge potentials before and after the 30-cycle durability test: (a) fresh electrode and (b) aged electrode.

**Table 4:** Fitted EIS Parameters of GDE_gma/PES before and after the 30-Cycle Aging/Durability Test

**Before** Discharge/Charge									
**potential (V vs RHE)**		***R***_**1**_ **(Ω)**	***R***_**2**_ **(Ω)**	***R***_**3**_ **(Ω)**	**CPE**_**1**_ **(mS·s** ^**a**^ **)**	***a***_**1**_	**CPE**_**2**_ **(mS·s** ^**a**^ **)**	***a***_**2**_	***W*** (S*s^**0.5**^ **)**
**discharged**	**0.3**	1.22 ± 0.14	0.28 ± 0.17	0.68 ± 1.15	1.40 ± 0.00	0.75 ± 0.34	684.20 ± 0.73	0.68 ± 0.34	1.76 ± 0.63
	**0.9**	1.32 ± 0.06	0.12 ± 0.24	0.27 ± 0.12	1072 ± 3.04	0.59 ± 0.94	0.35 ± 0.00	0.94 ± 0.28	0.84 ± 0.09
**charged**	**1.6**	1.15 ± 0.01	6.92 ± 0.94		289.60 ± 0.02	0.50 ± 0.02			
	**1.7**	1.18 ± 0.01	2.61 ± 0.18		247.30 ± 0.02	0.53 ± 0.02			

After Discharge/Charge									
**potential (V vs RHE)**		***R***_**1**_ **(Ω)**	***R***_**2**_ **(Ω)**	***R***_**3**_ **(Ω)**	**CPE**_**1**_ **(mS·s** ^**a**^ **)**	***a***_**1**_	**CPE**_**2**_ **(mS·s** ^**a**^ **)**	***a***_**2**_	***W*** (S*s^**0.5**^ **)**
**discharged**	**0.3**	1.21 ± 0.04	6.55 ± 0.09		0.16 ± 0.00	0.85 ± 0.01			0.87 ± 0.11
	**0.9**	1.20 ± 0.03	7.22 ± 0.09		0.38 ± 0.00	0.89 ± 0.01			0.96 ± 0.15
**charged**	**1.6**	1.17 ± 0.01	2.60 ± 0.04		6.63 ± 0.00	0.87 ± 0.02			
	**1.7**	1.34 ± 0.02	1.14 ± 0.03		6.58 ± 0.01	0.77 ± 0.03			

The aging test also demonstrated that the improved cycling behavior of GDE_gma/PES is not directly associated with enhanced intrinsic electrocatalytic kinetics. Instead, the superior long-term performance primarily originates from improved interfacial stabilization, regulated electrolyte management, and mitigation of catalyst degradation processes. This interpretation is strongly supported by the ICP-OES results, where significantly lower Mn and Ni dissolution was observed for GDE_gma/PES compared to the bare electrode. The reduced metal-ion migration suggests that the conformal poly­(GMA)-coated PES interlayer likely acts as a protective barrier that suppresses catalyst dissolution and preserves ionic transport pathways during prolonged cycling. Overall, these results demonstrate that the improved long-term behavior of GDE_gma/PES originates predominantly from stabilization of the gas–liquid–solid interface rather than enhancement of intrinsic ORR/OER kinetics.

ICP-OES analysis was conducted to quantify the diffusion of metal ions from the GDE catalyst layers into the electrolyte during cycling. As shown in Table 5, the electrolyte in contact with GDE_gma/PES contained markedly lower Mn and Ni concentrations than those in contact with b-GDE and GDE_gma. These results are consistent with the interpretation that the PGMA_1-coated PES interlayer suppresses catalyst-derived metal migration into the electrolyte. This suppression is likely associated with the barrier function of the conformal poly­(GMA) layer, which limits direct exposure of catalyst particles to the bulk electrolyte and reduces dissolution kinetics under alkaline conditions.

**Table 5:** Concentration Ratio of Metal Ions in the Electrolyte of the Half-Cell Consisting of b-GDE and GDE_gma/PES after Charge/Discharge Process

	**concentration ratio of metals in electrolyte (%)**	
electrode	**Mn**	**Ni**
**b-GDE**	1.43	0.22
**GDE_gma/PES**	0.72	0.08

The charge–discharge cycling stability of Zn–air batteries assembled with b-GDE, GDE_gma, and GDE_gma/PES was further examined at a constant current density of 5 mA cm^–2^ (Figure 10). Although the GDE_gma configuration was included in the Zn–air battery performance evaluation as an intermediate control sample, aging test and ICP-OES measurements were limited to b-GDE and GDE_gma/PES due to the less stable and less reproducible electrochemical behavior of GDE_gma during prolonged cycling. Therefore, the detailed aging-focused characterization was performed on the baseline and optimized electrode architectures to ensure reliable mechanistic comparison. The polarization gap (Δ*E*) varies among the electrodes, reflecting differences in electrochemical reversibility and cumulative interfacial losses during cycling. The b-GDE electrode exhibits a moderate voltage gap (Δ*E* ≈ 0.795 V) that remains nearly constant with time, indicating relatively stable but limited electrochemical reversibility. In contrast, the GDE_gma electrode shows the highest polarization (Δ*E* ≈ 0.984 V), suggesting increased internal resistance and less favorable cycling behavior. Notably, the GDE_gma/PES electrode demonstrates the lowest polarization (Δ*E* ≈ 0.734 V), and the voltage gap gradually decreases during cycling, indicating gradual stabilization of the electrode/electrolyte interface.

**Figure 10:**
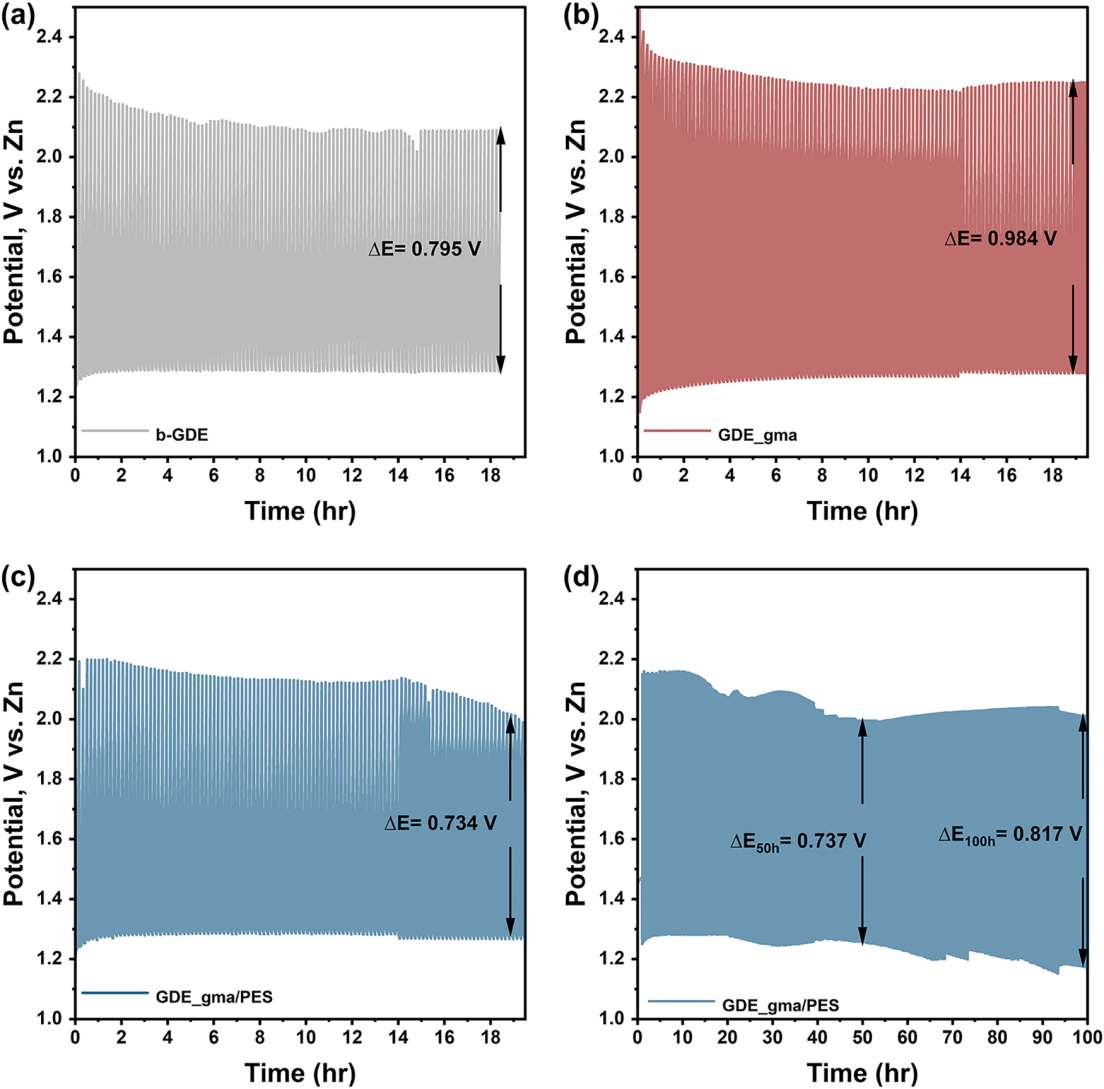
Extended galvanostatic charge–discharge cycling behavior of rechargeable Zn–air batteries employing (a) b-GDE, (b) GDE_gma, and (c) GDE_gma/PES air electrodes at 5 mA cm^–2^ in 6 M KOH electrolyte (d) long-term 100 h cycling evaluation of GDE_gma/PES. The voltage profiles illustrate the evolution of the charge–discharge polarization gap (Δ*E*) during cycling, providing insight into interfacial stability, degradation behavior, and practical electrode performance.

The extended galvanostatic cycling results further confirm that the improved long-term behavior of GDE_gma/PES originates primarily from enhanced interfacial stabilization and suppression of degradation processes rather than direct enhancement of intrinsic catalytic activity. The lower polarization growth observed during prolonged cycling is consistent with improved electrolyte management, preserved ionic transport pathways, and reduced catalyst dissolution within the modified architecture.

The charge/discharge energy efficiency reached 60.3% for GDE_gma/PES, compared with 57.6% for b-GDE. As shown in Figure 10, the b-GDE exhibits a nearly constant Δ*E* throughout cycling, indicating limited evolution of interfacial properties despite sustained operation. In contrast, the gradual decrease in voltage gap for GDE_gma/PES suggests progressive stabilization of the electrode/electrolyte interface and mitigation of cumulative polarization losses. This trend is consistent with ICP-OES results (Table 6), which confirm reduced metal dissolution and support the role of the poly­(GMA)-coated PES interlayer in preserving electrochemical integrity during prolonged cycling. Such behavior indicates that interfacial degradation processes dominate polarization in unmodified electrodes, whereas interfacial stabilization in GDE_gma/PES effectively mitigates these losses over time. The reduced polarization and improved efficiency of GDE_gma/PES highlight the importance of interfacial engineering in improving practical Zn–air battery operation. The GDE_gma configuration serves as an intermediate control sample, highlighting the necessity of the PES-supported interlayer for achieving stable and reproducible electrochemical behavior.

**Table 6:** Concentration Ratio of Metal Ions in the Electrolyte of the Zn-Air Battery Consisting of b-GDE and GDE_gma/PES after Charge/Discharge Process

	**concentration ratio of metals in electrolyte (%)**		
**electrode**	**Mn**	**Ni**	**Zn**
**b-GDE**	0.71	0.12	4.01
**GDE_gma/PES**	0.32	0.03	5.05

To further assess metal-ion dissolution during battery operation, the Zn^2+^ concentration in electrolytes after cycling was compared for Zn–air batteries containing b-GDE and GDE_gma/PES. As presented in Table 6, the electrolyte from the GDE_gma/PES cell exhibited a slightly higher Zn^2+^ concentration. This may be attributed to zinc passivation and ZnO precipitation accompanying increased Zn^2+^ accumulation, which could contribute to the slightly shorter cycling duration observed for GDE_gma/PES-containing Zn–air batteries. This further indicates that the optimized interfacial architecture plays a decisive role in controlling degradation pathways during battery operation.

Images of the electrolytes after cycling (see Figure S2 in the Supporting Information) further indicate that the PGMA_1-coated PES layer restricts metal-ion transfer from the air electrode into the electrolyte, even though Zn-containing species from the anode remain present in the cell.

SEM images of the gas diffusion electrode after charge–discharge cycling (see Figure S3 in the Supporting Information) show that the fibrous PES structure remains largely intact. Notably, the GMA coating is still visible on the fiber surfaces, indicating that the fibrous PES structure and conformal GMA coating remain largely preserved after prolonged electrochemical operation, supporting the structural stability of the interfacial architecture. The coating appears conformal and well adhered to the PES network, with no evident large-scale delamination or structural collapse.^47–49^

Considering the harsh alkaline environment and the mechanical stresses associated with oxygen evolution and electrolyte contact during cycling, the retained coating morphology suggests good chemical stability and interfacial adhesion of the GMA layer. This observation supports the conclusion that the PGMA_1-coated PES layer can function as a durable barrier within the GDE architecture while maintaining structural integrity after Zn–air battery operation.

The GDE_gma configuration, which lacks the PES-supported interlayer, exhibited less stable and less reproducible behavior during cycling. As a result, the cycling ICP-OES measurements showed significant variability, preventing reliable quantification of metal ion concentrations. Therefore, the comparison in Table 5 is limited to b-GDE and GDE_gma/PES, which provide more consistent and representative results. This approach allows clearer evaluation of the impact of the optimized interfacial architecture.

Overall, the results show that polymer-assisted surface functionalization improves the practical electrochemical behavior of gas diffusion electrodes by enhancing electrolyte management, limiting catalyst-derived metal dissolution, and stabilizing the electrode/electrolyte interface. The performance of GDE_gma/PES is therefore best understood as the combined consequence of improved ionic transport and reduced degradation, rather than as an unambiguous increase in intrinsic catalytic kinetics.

## 4 Conclusion

In this study, a solvent-free interfacial engineering strategy was developed to enhance the stability and electrochemical performance of gas diffusion electrodes (GDEs) in rechargeable Zn–air batteries. Using initiated chemical vapor deposition (iCVD), a dual-functional architecture consisting of a hydrophilic poly­(glycidyl methacrylate) interlayer and a hydrophobic poly­(2-(perfluorohexyl)­ethyl acrylate) outer coating was successfully integrated within the GDE structure. The hydrophobic PFHEA layer helps maintain stable gas transport pathways, prevents flooding of the gas diffusion layer, and preserves oxygen accessibility. The conformal poly­(GMA) coating significantly improved electrolyte affinity and ionic transport, leading to enhanced electrolyte uptake and a more than 2-fold increase in ionic conductivity compared with the unmodified electrode.

Electrochemical analyses showed that the GDE_gma/PES architecture improves interfacial transport and lowers charge–discharge polarization while suppressing catalyst and carbon migration into the electrolyte. Post-aging EIS and ICP-OES analyses further confirmed that the GMA-PES interlayer delays GDE degradation by preserving ionic pathways, suppressing catalyst-derived metal dissolution, and mitigating flooding-related transport losses. The improved performance of the modified electrodes is primarily governed by interfacial stabilization and transport enhancement rather than intrinsic catalytic activity. As a result, Zn–air batteries assembled with the modified electrodes exhibited improved round-trip energy efficiency and enhanced cycling stability. Importantly, ICP-OES and postcycling structural analyses support that the poly­(GMA)-coated PES interlayer functions as a durable barrier that mitigates metal-ion dissolution while maintaining structural integrity under alkaline operating conditions.

Overall, this work demonstrates that vapor-phase polymer deposition provides a versatile and scalable approach for engineering functional interfaces within porous battery electrodes. The integration of hydrophilic protective layers via iCVD offers an effective pathway to stabilize gas–liquid interfaces, regulate ion transport, and extend electrode durability. These findings highlight the potential of solvent-free polymer thin-film engineering as a promising strategy for the development of next-generation metal–air batteries and related electrochemical energy-conversion systems. Overall, this study emphasizes that interfacial engineering can decouple apparent catalytic activity from long-term electrode performance, providing a new design paradigm for durable gas diffusion electrodes in metal–air batteries.

## Supplementary Material



## Notes

Equivalent circuit models used for EIS fitting before and after cycling, photographs of electrolytes after cycling tests, postcycling SEM images of GDE_gma/PES electrodes, SEM images of Zn electrodes before and after cycling, and corresponding EDS analyses (PDF)
