# Supplementary material for: Solvent-Free Surface Engineering of Gas Diffusion Electrodes via Initiated Chemical Vapor Deposition for Durable Zinc–Air Batteries
Source: ACS Omega. 2026 Jun 30;11(27):40499–510. doi: 10.1021/acsomega.6c03441 (PMC13382815; doi:10.1021/acsomega.6c03441)
Supplement: Supplementary file 1 [file ao6c03441_si_001.pdf]

Solvent-Free Surface Engineering of Gas Diffusion Electrodes via Initiated Chemical Vapor Deposition for Durable Zinc–Air Batteries

Gizem CİHANOĞLU[1,], İklime KAYHAN[1,2], Özgenç EBİL[1,2]*

[1] Izmir Institute of Technology, Faculty of Engineering, Department of Chemical Engineering, Urla 35430, Izmir, Türkiye

[2] ADVENST Energy Storage Systems, Gulbahce Mah., Kuluçka Merkezi Binası No: 1/45/11 Urla 35430 Izmir, Türkiye

[*]Corresponding author:

E-mail: gizemcihanoglu@iyte.edu.tr

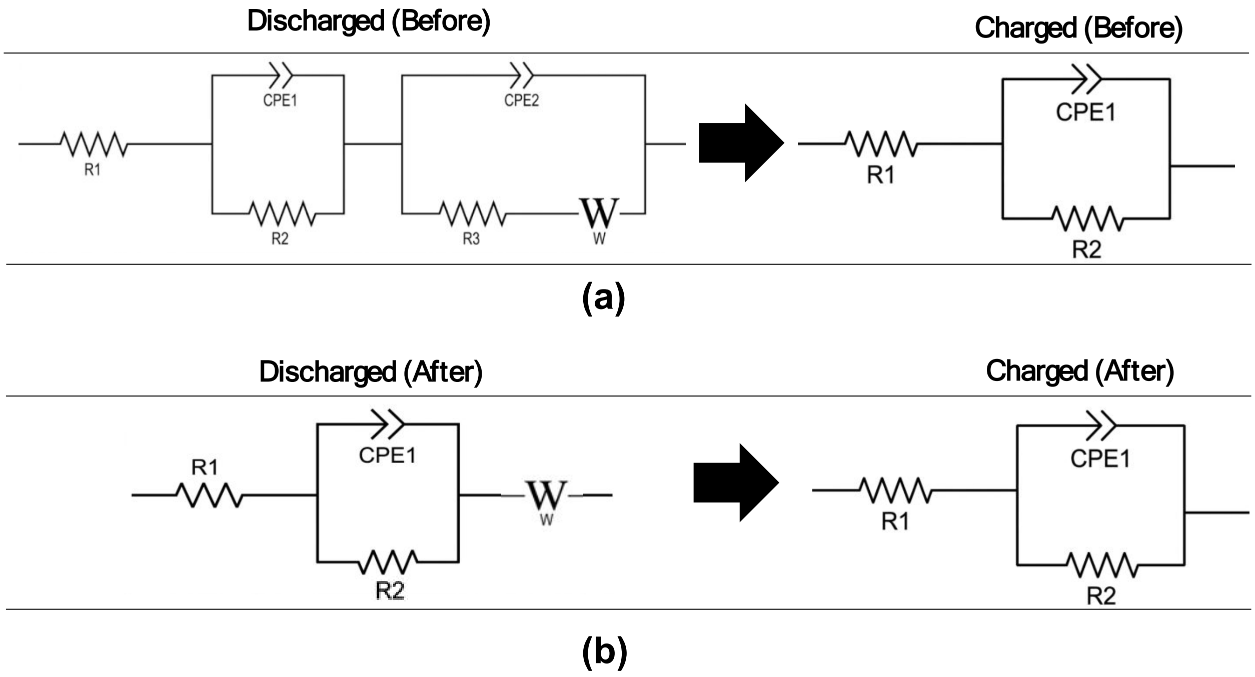

Figure S1. Equivalent Electrical Circuit Models for GDE_gma/PES electrodes (a) before and (b) after the discharge/charge test (Resistances (Ri), Constant phase element (CPE), Warburg impedance (W)).

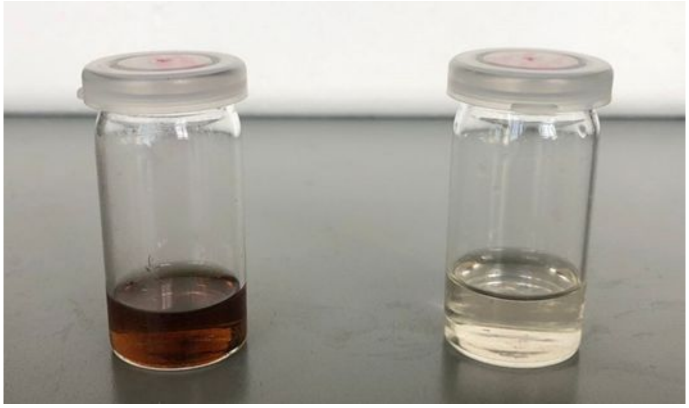

Figure S2. Images of 6 M KOH solution electrolyte after charge-discharge cycles of Zn-air batteries, including b-GDE (left), and GDE_gma/PES (right)

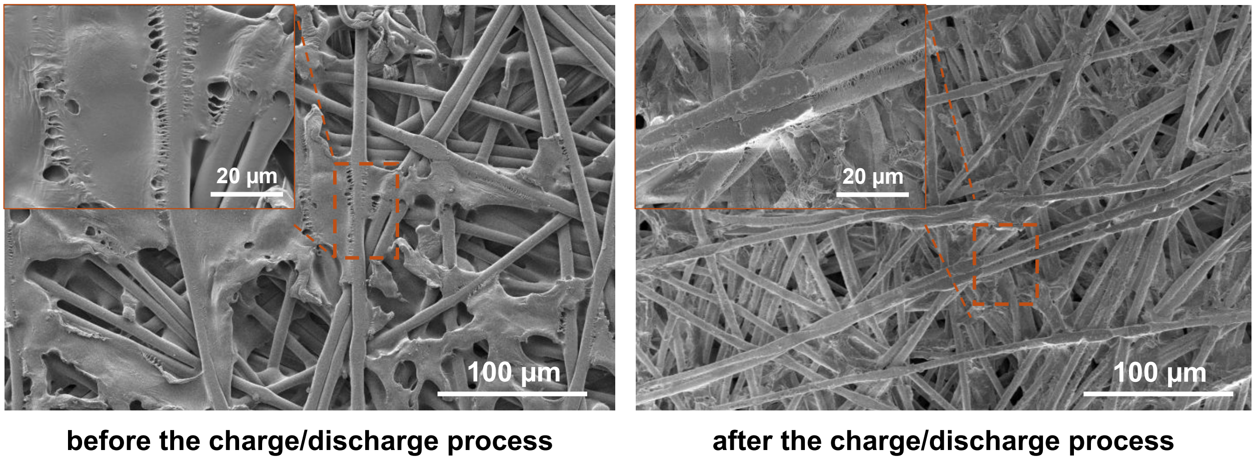

Figure S3. The SEM images of GMA-coated PES before and after charge/discharge process

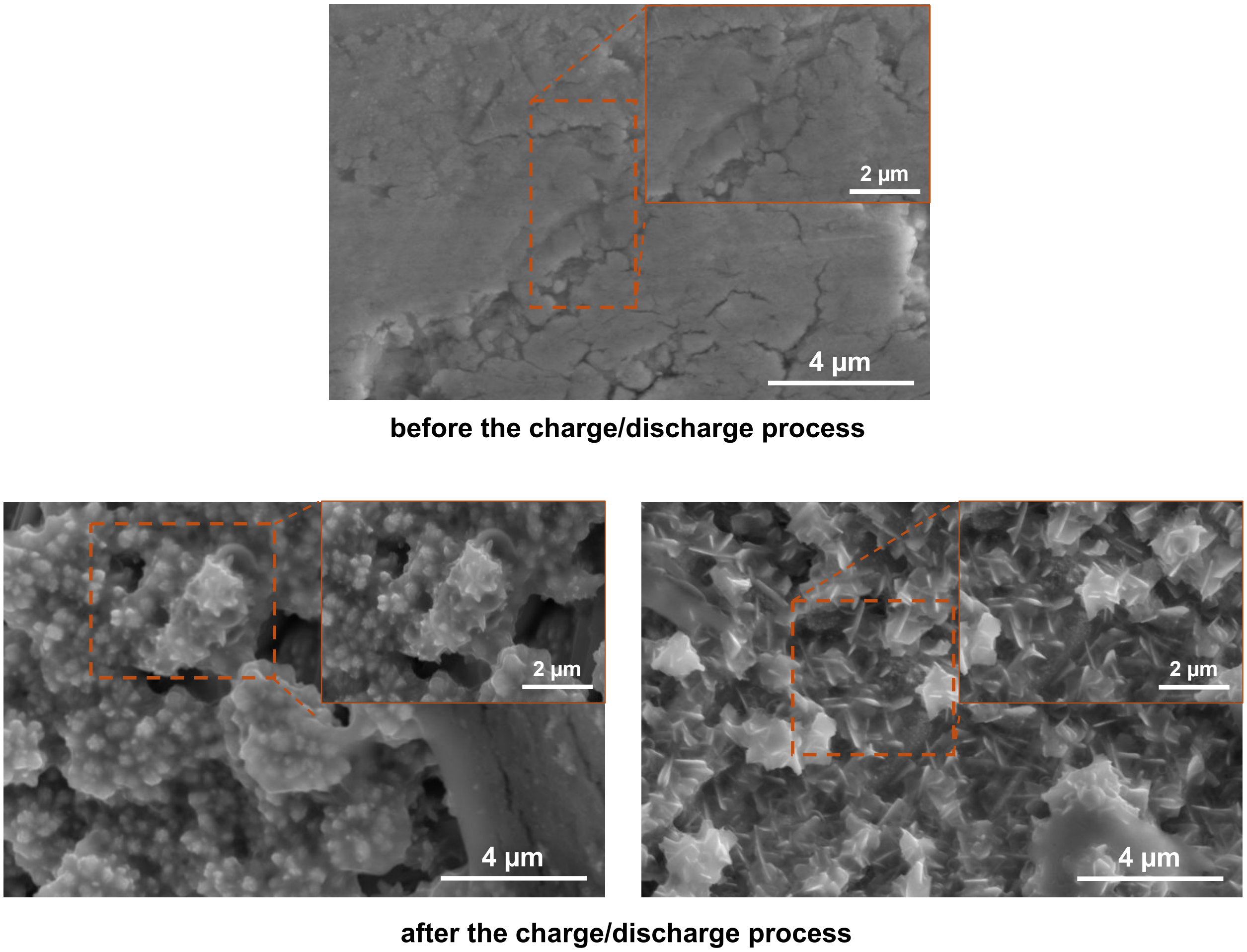

Figure S4. The SEM images of Zn plate before and after charge/discharge process

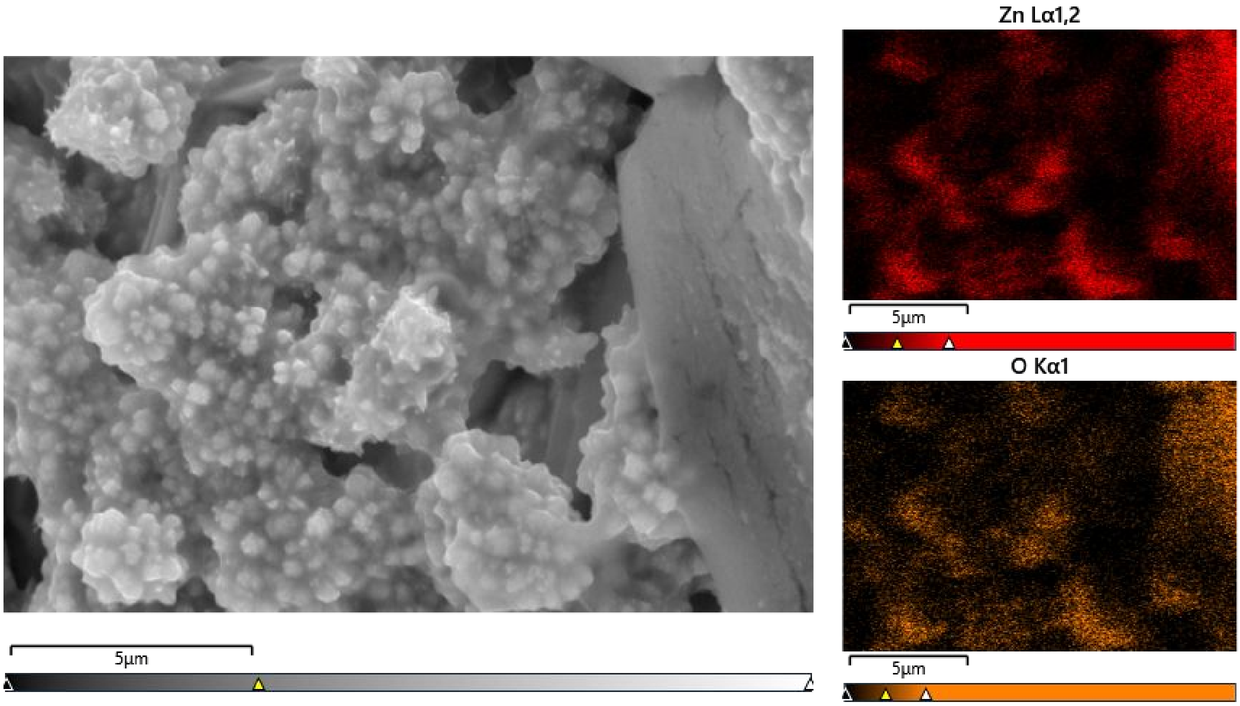

Figure S5. The EDS analysis of Zn plate before and after charge/discharge process
